# Extracorporeal Membrane Oxygenation Use in Thoracic Surgery

**DOI:** 10.3390/membranes11060416

**Published:** 2021-05-31

**Authors:** Pavel Suk, Vladimír Šrámek, Ivan Čundrle

**Affiliations:** 1 International Clinical Research Center, St. Anne’s University Hospital Brno, 65691 Brno, Czech Republic; 2 Department of Anesthesiology and Intensive Care, St. Anne’s University Hospital Brno, 65691 Brno, Czech Republic; vladimir.sramek@fnusa.cz; 3 Faculty of Medicine, Masaryk University, 62500 Brno, Czech Republic

**Keywords:** extracorporeal membrane oxygenation, thoracic surgery, lung resection, tracheal resection, carinal resection

## Abstract

This narrative review is focused on the application of extracorporeal membrane oxygenation (ECMO) in thoracic surgery, exclusive of lung transplantation. Although the use of ECMO in this indication is still rare, it allows surgery to be performed in patients where conventional ventilation is not feasible—especially in single lung patients, sleeve lobectomy or pneumonectomy and tracheal or carinal reconstructions. Comparisons with other techniques, various ECMO configurations, the management of anticoagulation, anesthesia, hypoxemia during surgery and the use of ECMO in case of postoperative respiratory failure are reviewed and supported by two cases of perioperative ECMO use, and an overview of published case series.

## 1. Introduction

The securing of adequate ventilation may be problematic in some lung surgery procedures. Firstly, the provision of adequate gas exchange in patients after previous lung resections or with significant lung impairment may be difficult. Secondly, surgical procedures on the trachea and/or carina may result in a collision between the airway tube and surgical field.

In post-pneumonectomy patients, surgery on a ventilated lung, high-frequency jet ventilation (HFJV) or apneic oxygenation is possible [1]. However, surgery on a ventilated lung is difficult, uncomfortable for the surgeon and complex surgical procedures are frequently not feasible. Apnea with high-flow oxygen may lead to severe hypercapnia with respiratory acidosis. Similar situations occur in patients with chronic (e.g., chronic obstructive pulmonary disease) or acute respiratory failure (e.g., pneumonia, acute respiratory distress syndrome or trauma), when one-lung ventilation—even in combination with continuous positive airway pressure (CPAP) or HFJV on a non-dependent lung—cannot secure hematosis.

In carinal and tracheal reconstruction surgery, high-frequency jet ventilation or periodic cross-field intubations alternated with periods of apnea are feasible [2]. However, these techniques have several limitations, which include difficulties in securing airways in patients with severe trachea-carinal stenosis, hypercapnia during interrupted ventilation and HFJV and hemodynamic instability in the case of heart maneuvers [3]. Distal airway pressure monitoring with a dual-lumen catheter reduced the risk of barotrauma; however, HFJV presents a threat of mucosal tumor cell spread. Moreover, frequent manipulations of the tracheal tube may worsen surgical conditions and require excellent cooperation between the surgeon and anesthetist.

Limitations of the above-mentioned ventilation strategies have led to the introduction of extracorporeal devices in lung resection surgery, including cardiopulmonary bypass (CPB), extracorporeal CO_2_ removal (ECCO_2_R) and extracorporeal membrane oxygenation (ECMO).

## 2. Cardiopulmonary Bypass

CPB provides full respiratory and hemodynamic support. CPB is required when the cardiac cavities are opened and during surgery involving large thoracic vessels. A venous reservoir of CPB allows the re-transfusion of suctioned blood and the administration of drugs and fluids. However, the use of CPB has several disadvantages. Direct blood–air contact strengthens the activation of coagulation and inflammation, which necessitates the administration of high-dose heparin. The use of a venous reservoir increases the priming volume, which leads to hemodilution. Moreover, the re-entry of suctioned blood into the vascular system may present a risk for tumor dissemination [4,5], although this has not been confirmed by other authors [6].

To reduce CPB disadvantages, a minimally invasive extracorporeal circulation (MiECC) was developed. The venous reservoir is replaced with a bubble trap or an air-removing device inserted into the venous line to reduce the risk of air embolism. This biocompatible closed-circuit system without blood–air contact leads to the reduction of priming volume, fewer blood transfusions and decreased inflammatory response in comparison to conventional CPB [7]. However, the use of MiECC allows only a moderate reduction of heparin doses.

ECMO has the simplest circuit setup without any components in the venous line, which allows a significant reduction or omission of anticoagulation. This advantage was demonstrated with an example of lung transplantation, because patients on ECMO required fewer transfusions and had less bleeding and fewer reoperations than patients on CPB [8]. Moreover, some of the procedures previously reserved for the use of CPB (left atrium or descending aorta surgery) were successfully performed using veno-arterial (VA) ECMO [9].

## 3. Extracorporeal CO_2_ Removal

The simplest method of extracorporeal gas exchange is pumpless interventional lung assist (iLA) or pumpless ECCO_2_R. In modern membrane oxygenators, blood flows outside the mesh of crossed hollow fibers while sweep gas flows inside the fiber lumens. Since only 25% of cardiac output is required to pass through the oxygenator to eliminate CO_2_ [10], the large cross-sectional area and low hydraulic resistance oxygenator, together with short tubing, allow sufficient blood flow (1.2–2.2 L/min) driven by the difference between arterial and venous pressure [11,12]. A drainage catheter is introduced into the femoral artery, and blood is returned to the femoral vein. Although this method is simple and easy to use, inadequate oxygenation and dependence on the patient’s cardiac output limits the use of interventional lung assist for selected patients without impaired oxygenation and good cardiac function [3].

The evolution of iLA is veno-venous (VV) ECCO_2_R, which primarily reduces complications related to arterial cannulation. A single venous double-lumen cannula is introduced into the internal jugular or femoral vein, and blood flow is driven by a centrifugal pump with similar blood flow to the AV setup [12]. Both ECCO_2_R variants are suitable for patients undergoing lung surgery when lung disease or previous lung resection(s) present a risk for hypercapnia with protective one-lung ventilation. The broadest experience is with lung volume reduction surgery in either AV [11] or VV [13] setup. Moreover, ECCO_2_R in combination with apneic oxygenation provides full respiratory support [14] and extends the indications to procedures with short-term apnea.

## 4. Extracorporeal Membrane Oxygenation

ECMO can provide either partial respiratory support with preserved one-lung ventilation or full respiratory support without complicated airway management during surgery. The major advantages are excellent conditions for a surgeon in combination with preserved gas exchange on lung-protective ventilation or without ventilation at all. Moreover, ECMO is designed for prolonged use and therefore may be continued postoperatively. Consequently, ECMO is preferred to CPB and/or ECCO_2_R in most lung resections and complex airway procedures. In the remainder of this review, ECMO configuration and perioperative use is discussed, introduced here with two cases of typical perioperative VV ECMO indications in our institution.

### 4.1. Case 1

**Figure 1:**
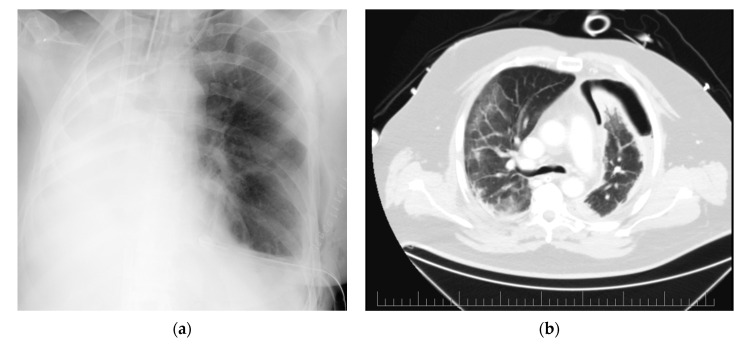
Radiographic images of presented cases: (**a**) Postoperative chest X-ray of a patient after left-sided double atypical resection and previous right-sided pneumonectomy (ECMO cannulas introduced via right internal jugular and femoral veins); (**b**) Preoperative CT scan of a patient with bilateral COVID-19 pneumonia with a ground glass and crazy paving pattern and left-sided pneumothorax.

Firstly, we present a 59-year-old woman with a diagnosis of melanoma. Three years after resection of the primary tumor, she underwent uncomplicated right-sided pneumonectomy due to a metastasis revealed in the right pulmonary hilus. Four years later, another two lesions were found in the left lower lobe. Several treatment options were offered to the patient, and she strongly preferred another lung resection. On the day of surgery, the patient was anesthetized and intubated, and protective mechanical ventilation was initiated. After anticoagulation with 2000 IU of unfractionated heparin, a 23 French venous cannula was placed in the right femoral vein and a 17 French outflow cannula was placed in the right internal jugular vein under ultrasound control. ECMO was initiated with 2 L/min of blood flow with 2 L/min of sweep gas and an oxygen fraction of 50%. As soon as the chest cavity was opened, mechanical ventilation was stopped. ECMO blood flow was then increased to 4 L/min and sweep gas to 4 L/min with an oxygen fraction of 50%. After the removal of the pleural adhesions, a double atypical wedge resection was carried out. The surgery was uncomplicated and lasted 2 h and 30 min, during which the patient was on full ECMO support without mechanical ventilation. After the surgery, a chest X-ray was taken (Figure 1a), and 4 h later the patient was extubated and weaned off ECMO. Her stay at hospital was uncomplicated, and she was discharged 11 days after the surgery.

### 4.2. Case 2

In the second case, we present a 56-year-old man admitted to the ICU with severe COVID-19 pneumonia. The patient was intubated and mechanically ventilated from day 3. On day 11, the patient’s status rapidly deteriorated because of a left-sided tension pneumothorax (PTX). A chest tube was inserted with an initial air-leak of 3 L/min. The air-leak was persistent over the several next days. A thoracic surgeon was then consulted, and the chest CT revealed bilateral pneumonia (with a ground glass and crazy paving pattern) and left-sided PTX with air capsules in the lingual lobe (Figure 1b). As a result, a revision of the left pleural cavity was indicated.

The patient was unable to undergo one-lung ventilation, and perioperative VV ECMO support was indicated. After the administration of heparin 2000 IU, a 25 French venous cannula was placed in the right femoral vein and a 19 French outflow cannula was placed in the right internal jugular vein. VV ECMO (blood flow of 4 L/min, gas sweep of 8 L/min) and protective ventilation of the right lung were initiated. The source of the air leak was a ruptured abscess in the lingula, which was resected using a buttress stapler. The patient remained postoperatively on ECMO without an air leak. However, on day 19, the air leak reappeared (1.5 L/min), and a surgical revision was indicated. A bronchial fistula was found in the necrotic part of the primary lesion. An atypical resection of the necrotic lobe was performed, and tissue glue was used on the resection line. There was no air leak at the end of the surgery. The patient was left on one-lung ventilation to increase the fistula’s chance of remaining sealed. VV ECMO support was 4.5 L/min of blood flow with sweep gas of 10 L/min to maintain acceptable blood gases. On day 21, the protective ventilation of the left lung was restarted, with no signs of air-leak. A repeated CT showed no signs of PTX on day 25. The patient’s condition continued to improve, and he was successfully weaned off ECMO on day 29 of his ICU stay. On day 41, the patient was successfully weaned off mechanical ventilation and on day 42 he was discharged from the ICU. The patient was discharged home on day 97.

### 4.3. ECMO Configurations and Indications

The configuration closest to CPB is VA ECMO. It provides full respiratory support (O_2_ delivery and CO_2_ removal even during apnea) as well as circulatory stability in cases in which manipulations of the heart and aorta are necessary—e.g., carinal resection by sternotomy [15,16]; in patients with limited cardiac function or severe pulmonary hypertension. Peripheral or central cannulation are both feasible, with the latter usually reserved for procedures by sternotomy. VA ECMO can be converted to a VV configuration for postoperative respiratory support. However, VA ECMO has many disadvantages including a risk of systemic embolization, arterial dissection and thrombosis, differential upper body hypoxia (Harlequin syndrome) and limb ischemia in the case of peripheral cannulation. Furthermore, surgical artery repair or the use of a vascular closure device is commonly necessary during arterial cannula removal.

VV ECMO secures full respiratory support without influencing the circulation. In case of hemodynamic instability, VV ECMO can be converted to a VA or veno-arterial-venous configuration. VV ECMO has no artery-related complications but the proximity of suction and outflow cannula tips (or openings on dual-lumen cannula) presents a risk for recirculation, which decreases the effective blood flow. High-flow dual-lumen cannulas are not commonly used perioperatively because it is difficult to maintain an ideal location during patient positioning and surgery. An optimal cannulation strategy for VV ECMO appears to be drainage from the upper inferior vena cava (IVC) with a return of oxygenated blood into the superior vena cava [17] or vice versa [18]. The recirculation can also be diminished by using a multistage suction cannula, which has side holes spread over 10 to 20 cm from the tip and drains blood predominantly from the proximal holes [19]. When the cannulation of the internal jugular vein on the neck is impractical due to trauma, subcutaneous emphysema or interference with a surgical field, both cannulas can be introduced through femoral veins: a multistage suction cannula into the lower IVC with the tip situated at the level of the renal veins, and a single stage outflow cannula to the right atrium [20].

The hollow fiber membranes of ECMO oxygenators are mostly made of polymethylpentene (PMP). This non-microporous membrane is minimally water-permeable and prevents plasma leakage and, therefore, allows prolonged use, albeit at a higher cost. Although polypropylene membranes with micropores suffer from plasma leakage, it is sufficient for intraoperative use in patients with a low risk of postoperative ECMO support.

VA ECMO is usually limited to procedures with heart manipulations and patients with acute or chronic heart dysfunction or severe pulmonary hypertension. The typical procedures are carinal surgery by sternotomy and a combination of lung resection(s) with an atrial or aortic resection. VV ECMO provides full respiratory support; therefore, common surgical procedures include lung resections in patients with a single lung or with limited function of their non-operated lungs, resections of trachea, carina or mainstem bronchus. An overview of case series including more than four patients is presented in Table 1 (232 patients in total).

## 5. Management of Anticoagulation

ECMO therapy leads to contact between circulating blood and the foreign surface of the extracorporeal circuit, which activates inflammatory and coagulation responses. The development of more biocompatible materials decreases this interaction. Heparin-bonded circuits (HBC)—the most prevalent type of coating—reduced transfusion requirements, activation of white blood cells and complement, and reduced length of stays in the ICU, as proved by a meta-analysis [26]. Although the effect of HBC was mainly limited to cardiac surgery and 6-h circuit use, these findings are important for perioperative use. Nevertheless, other studies proved no benefit from the heparin coating of CPB [27], or only a short-term effect (limited to 6 h) in the case of ECMO [28]. Although the effect of HBC on longer circuit-runs is not clear, HBC circuits are routinely used in the majority of ECMO reports.

In thoracic surgery, heparin administration needs to be reduced due to the risk of bleeding. In French centers [16], 50–100 IU/kg of heparin was administered before cannulation with subsequent heparin titration to reach ACT between 160 and 200 s—an only slightly reduced level in comparison with ELSO guidelines. Although heparin was withheld in case of bleeding, 6 of 36 patients were reoperated for bleeding at the operating site. Similarly, the target-activated partial thromboplastin time (APTT)-ratio of 1.5 during surgery was associated with reoperation in 2 of 24 patients [9]. Two other centers opted for a more conservative approach: a bolus of heparin 3000–5000 IU intravenously before cannulation without any further heparin [22,29]. Similarly, another center in Korea used a 50–70 IU/kg heparin bolus immediately before the insertion of the cannulas, withheld anticoagulation during and for 24 h after surgery and subsequently adjusted heparin with the target ACT of 160–190 s [25]. No major thrombotic events were reported in these studies. Therefore, omitting heparin during and shortly after surgery appears to be safe.

Furthermore, despite some manufacturers’ recommendations, the circuit can be primed with a sterile isotonic electrolyte solution as recommended by the ELSO Anticoagulation Guidelines. An addition of heparin to the priming fluid can increase the risk of bleeding during surgery.

## 6. Anesthesia

Despite some limitations of drug delivery and monitoring [30], volatile anesthetics are regularly used in more than a third of centers in patients on CPB [31]. Although critically ill patients with ARDS on ECMO can be sedated with inhaled isoflurane using the AnaConDa system [32,33], ECMO devices are not designed for the delivery of volatile anesthetics. Since ventilation is interrupted during the procedure, inhalational anesthetics cannot be reliably used.

Intravenous anesthetics are lipophilic drugs that cross the blood–brain barrier. However, the associated high protein binding is related to adsorption on the circuit [34]. A decrease of approximately 50% in the plasma concentrations of midazolam and fentanyl was observed over 1 h in vitro, while morphine concentration did not change [35,36]. Propofol absorption was even faster—the plasma concentration halved in approximately 20 min [37,38], and this change was dependent on the propofol concentration but not on the circuit coating. Interestingly, the adsorption to polyvinyl chloride tubing and contact with oxygen were responsible for most of these losses [35]. Neuromuscular blocking agents are hydrophilic, with a low expected circuit adsorption. Pharmacokinetic changes of other drugs used in perioperative care are summarized in a review by Cheng et al. [39].

The extracorporeal circuit does not significantly increase the distribution volume but together with surgery may change blood flow through the liver and kidney and, thus, decrease drug clearance. Based on both unpredictable drug concentrations and a poor correlation between plasma concentrations and anesthesia depth, continuous monitoring of anesthesia depth with the bispectral index (BIS) or a similar monitor is reasonable. Moreover, the assessment of neuromuscular transmission with a peripheral nerve stimulator should be utilized.

In most cases, cannulation and the initiation of ECMO is carried out after the induction of general anesthesia. This provides comfort for the patient and the cannulation itself. Awake cannulation is necessary in cases of severe airway stenosis when intubation is postponed after airway stenosis repair [29] or expected hemodynamic instability.

## 7. Hypoxemia during Surgery

In patients on VV ECMO, more than 75% of cardiac output (approx. 50 mL/min/kg of adjusted body weight) must pass the oxygenator to maintain arterial SpO_2_ at approx. 90% during apnea. Therefore, it is necessary to select cannulas which allow sufficient blood flow. If an adequate effective ECMO blood flow cannot be reached, the aim is to decrease cardiac output in order to increase the proportion of oxygenated blood on cardiac output. Intravenous beta-blockers and alpha-2-agonists are the most suitable drug classes. Nonetheless, this intervention is usually limited by hypotension and decreased O_2_ delivery.

When VV ECMO is not able to provide sufficient oxygen saturation, lung ventilation must be augmented. Since CO_2_ elimination is usually not an issue, intermittent (one-lung) ventilation does not present a major advantage over CPAP or apneic oxygenation. The effective usage of a small flexible catheter (e.g., suction catheter), inserted into the trachea or main bronchus with an O_2_ flow of 2–10 L/min, was demonstrated by Keeyapaj and Alfirevic [40]. Using a narrow catheter helps to maintain a clear operation field and prevents airway obstruction, which may lead to lung overinflation and barotrauma. HFJV may offer another alternative.

For VA configuration with peripheral cannulas, the main issue may be hypoxemia of the upper body (Harlequin syndrome), which receives poorly oxygenated blood passing through the lungs and the heart. For its detection, SpO_2_ and arterial blood gases must be obtained from the right upper extremity. Adequate PEEP and higher FiO_2_ must be used to prevent this complication. During apnea, CPAP, apneic oxygenation or HFJV may improve oxygenation in the lungs. When these measures fail, switching from peripheral to central cannulation is necessary.

## 8. Postoperative Management

Although the incidence of postoperative ARDS in thoracic surgery patients is below 3% [41,42], it rises to almost 8% in cases of pneumonectomy. Postoperative respiratory failure is associated with a poor outcome (mortality 25–50%). Therefore, it seems ideal to wean patients off the ventilator and ECMO support as soon after surgery as possible. However, in patients who have undergone repeated lung resections or with perioperative respiratory failure, fast weaning may not be possible. In contrast to CPB, both VV and VA ECMO support may be prolonged after the surgery to maintain lung-protective ventilation. Moreover, provided there is stable circulation, VA ECMO can be converted to the VV configuration.

It is not clear whether a patient should first be weaned off mechanical ventilation, or ECMO. A case series of ten awake patients on ECMO due to post-operative ARDS—seven of them after thoracic procedure—reported 80% survival, which supports earlier weaning off the ventilator [43]. Furthermore, there are several case reports on the use of VV ECMO following pneumonectomy due to trauma [44] or oncologic surgery [45] with good outcomes.

## 9. Conclusions

The use of extracorporeal support allows the safe carrying out of thoracic surgery procedures in selected patients who would otherwise be inoperable. It provides adequate gas exchange with excellent conditions for a surgeon despite patient- or surgery-related limitations. Several options—including CPB, ECMO and ECCO_2_R—are available, each of them burdened with specific disadvantages. Over time, VV ECMO seems to be the best option for either partial or full respiratory support, except for procedures involving heart manipulations or patients with heart failure, which require VA ECMO for combined respiratory and cardiac support.

## Figures and Tables

**Table 1:** Case series describing at least 5 patients undergoing thoracic or airway reconstruction procedure on ECMO or ECCO_2_R.

Author, Year	Indications for ECMO or ECCO_2_R	Prevalent Types of Surgery	Number of Patients (Configuration: Cannulation)	Intraoperative Heparin	Time on ECMO or ECCO_2_R	ECMO-Related Complications	Hospital/30-Day Mortality
Wiebe K, 2010 [11]	6 single lung 4 LLF due to ARDS	5 tracheal resection or repair 4 lung resection 1 partial decortication	10 (pumpless ECCO_2_R: V 19F fem-A 17F fem)	can 500–1000 IU none	6 intraop. 4 postop. for 6.8 days	1 retroperitoneal hematoma	2/10 (20%) in total 0/4 (0%) elective 2/6 (33%) urgent
Chang X, 2014 [21]	7 CTBR	7 tracheal resection	7 (VA: V 19F fem-A 17F fem)	can 200 IU/kg ACT 300 s	10–31 min	none	0/7 (0%)
Redwan B, 2015 [14]	5 single lung 3 LLF 1 carinal resection	7 pulmonary resection 2 extended metastasectomy 1 carinal PE	9 (3 VV: V 25F fem-V 21 IJV; 6 ECCO_2_R: 24 F DL fem)	2000–4000 IU none	129 ± 40 min intraop. only	not reported	1/9 (11%)
Lang G, 2011 [15] Lang G, 2015 [22]	8 CTBR 2 LLF	6 carinal resection 3 sleeve (bi)lobectomy 1 sleeve PE	10 (VA: 7 central RA-AoA + 3 peripheral fem-fem)	can 3000–5000 IU none	113 ± 17 min intraop. only	none	0/10 (0%)
Rinieri P, 2015 [16]	23 CTBR 5 single lung 5 LLF including trauma 3 ECMO preoperatively	tracheal/carinal reconstruction, wedge resection to PE, lymphadenectomy	36 (16 VA: 6 central + 10 peripheral; 20 VV; 5 ECCO_2_R)	can 50–100 IU/kg ACT 160–200 s	median 65 min for VA and 78 min for VV ECMO, many postop.	7 reoperations due to bleeding (6 operation site, 1 cannulation site)	6/36 (17%) in total 1/27 (4%) elective 5/9 (56%) urgent
Kim SH, 2017 [23]	6 post-intubation or post-tracheostomy stenosis	tracheal/carinal reconstruction	9 (1 VA: peripheral; 8 VV: 6 fem-fem + 2 fem-IJV)	can 50–100 IU/kg ACT 150–180 s	7 intra-op. for 1.5–4 h, 2 postop	none	1/9 (11%) 0/6 elective 1/3 (33%) urgent
Akil A, 2020 [13]	65 emphysema with hypercapnia	65 LVRS	65 (ECCO_2_R: 24F DL fem or 22F DL IJV)	none	all postop., mean 3 days	1 disseminated intravascular coagulopathy	90-day mortality 5/65 (8%)
Kim CW, 2015 [24] Kim DH, 2021 [25]	27 LLF (pneumonia etc.) 19 airway disease	27 lung resection 19 airway surgery 17 others	63 (21 VA: peripheral fem-fem; 42 VV fem-IJV)	can 50–70 IU/kg none	mean 4.5 days postop.	not reported	17/63 (27 %) in total 9/11 (82%) eCPR
Koryllos A, 2021 [9]	8 CTBR 16 left lung and descending aorta or left atrium resection	sleeve lobectomy or PE, lobectomy or PE with left atrial or aortic resection	24 (7 VA fem-fem; 9 VV-A fem+IJV-fem; 8 VV: 7 fem-IJV + 1 fem-fem)	APTT-R 1.5	not reported	2 reoperations due to bleeding at operation site	6/24 (25%) mortality of 4 acute cases not reported

Legend: A—arterial, ACT—activated clotting time, AoA—ascending aorta, APTT—activated partial thromboplastin time, can—dose administered before cannulation, CTBR—complex tracheobronchial reconstructions, DL—double lumen cannula, ECCO_2_R—extracorporeal CO_2_ removal, ECMO—extracorporeal membrane oxygenation, eCPR—extracorporeal cardiopulmonary resuscitation, F—French, fem—femoral, IJV—internal jugular vein, LLF—limited lung function, LVRS—lung volume reduction surgery, PE—pneumonectomy, RA—right atrium, V—venous; numbers at the beginning of a line represent the number of patients (if stated).
